# Targeted nanoparticles modify neutrophil function *in vivo*

**DOI:** 10.3389/fimmu.2022.1003871

**Published:** 2022-10-05

**Authors:** Sandra Völs, Naomi Kaisar-Iluz, Merav E. Shaul, Arik Ryvkin, Haim Ashkenazy, Avishag Yehuda, Ronza Atamneh, Adina Heinberg, Meital Ben-David-Naim, Menucha Nadav, Shira Hirsch, Vera Mitesser, Seth J. Salpeter, Ron Dzikowski, Zvi Hayouka, Jonathan M. Gershoni, Zvi G. Fridlender, Zvi Granot

**Affiliations:** ^1^ Department of Developmental Biology and Cancer Research, Institute for Medical Research Israel Canada, Hebrew University Medical School, Jerusalem, Israel; ^2^ Faculty of Medicine, Hebrew University of Jerusalem, Jerusalem, Israel; ^3^ Institute of Pulmonary Medicine, Hadassah-Hebrew University Medical Center, Jerusalem, Israel; ^4^ The Shmunis School of Biomedicine and Cancer Research, George S. Wise Faculty of Life Sciences, Tel Aviv University, Tel Aviv, Israel; ^5^ Institute of Biochemistry, Food Science and Nutrition, The Robert H. Smith Faculty of Agriculture, Food and Environment, The Hebrew University of Jerusalem, Rehovot, Israel; ^6^ Immunyx Pharma, Jerusalem, Israel; ^7^ Department of Microbiology and Molecular Genetics, Kuvin Center for the Study of Infectious and Tropical Diseases, Institute for Medical Research Israel-Canada, Hebrew University Hadassah Medical School, Jerusalem, Israel

**Keywords:** neutrophil, inflammation, nanoparticles, targeted delivery, reactive oxygen species

## Abstract

Neutrophils play critical roles in a broad spectrum of clinical conditions. Accordingly, manipulation of neutrophil function may provide a powerful immunotherapeutic approach. However, due to neutrophils characteristic short half-life and their large population number, this possibility was considered impractical. Here we describe the identification of peptides which specifically bind either murine or human neutrophils. Although the murine and human neutrophil-specific peptides are not cross-reactive, we identified CD177 as the neutrophil-expressed binding partner in both species. Decorating nanoparticles with a neutrophil-specific peptide confers neutrophil specificity and these neutrophil-specific nanoparticles accumulate in sites of inflammation. Significantly, we demonstrate that encapsulating neutrophil modifying small molecules within these nanoparticles yields specific modulation of neutrophil function (ROS production, degranulation, polarization), intracellular signaling and longevity both *in vitro* and *in vivo*. Collectively, our findings demonstrate that neutrophil specific targeting may serve as a novel mode of immunotherapy in disease.

## Introduction

Neutrophils are short lived polymorphonuclear granulocytic myeloid cells, the most abundant white blood cell in the human circulation. They represent the first line of defense against microbial infections and play a critical role in inflammatory processes. Their differentiation occurs in the bone marrow, yielding short-lived cells (1). It is estimated that every human produces ~1 billion neutrophils per kg daily at steady state, and up to 10 times more under inflammatory conditions (2). Notably, neutrophils from the circulation can rapidly migrate into sites of infection or inflammation (3).

Traditionally, neutrophils were considered to be a homogeneous population of terminally differentiated cells with limited functional plasticity. However, there is an increasing body of evidence to suggest that neutrophils are a diverse and plastic population of cells with varied functions (4–6). The main tasks of neutrophils are related to their anti-microbial activity. As such they are capable of phagocytosis, secretion of reactive oxygen species (ROS), proteases and nitric oxide (NO), autophagy and NETosis (7). Indeed, neutrophils possess granules containing antimicrobial compounds, but also additional proteases, lysozyme, metalloproteases, and mediators of the oxidative burst (8, 9). Some of the additional functions that neutrophils possess are direct cytotoxicity to cells (mostly *via* ADCC), the suppression, recruitment and modulation of other immune cells (6, 7, 10).

Although neutrophil cytotoxicity is beneficial for fighting infections, in many other pathological and chronic conditions these activities can cause significant collateral damage thereby aggravating pathological conditions (11–13). In addition, the notion that neutrophils are massively recruited to sites of sterile injury have raised the possibility that they have an active role in tissue repair (14). An important aspect of neutrophils’ capabilities that is being described is their many effects on the adaptive immune response. Neutrophils were found to be capable of modulating adaptive reactions *via* the production of cytokines, and even direct interactions with lymphocytes and dendritic cells (6).

Using discrete parameters (e.g., localization, shape, surface markers and more), heterogeneous populations of circulating and tissue residing neutrophils have been described in health and in different states of disease including infections, autoimmune & inflammatory disorders and cancer (15–18). Importantly, there is no clear consensus criteria to reproducibly and unequivocally define clinically relevant distinct neutrophil subsets. In the circulation, the two main sub-populations of neutrophils described by us and others include normal density neutrophils (NDN) and low-density neutrophils (LDN) (19). Other important sub-populations described include tissue residing neutrophils such as tumor-associated neutrophils, that have been further suggested to divide to N1 and N2 neutrophils (20, 21).

Neutrophils can also play a conflicting role in a wide variety of clinical conditions (7). Some of the non-infectious diseases in which major involvement of neutrophils in the pathogenesis has been described include inflammatory diseases such as Inflammatory bowel diseases (IBD) (22), Adult respiratory distress syndrome (ARDS) (23) and Chronic Obstructive Pulmonary diseases (COPD) (24), as well as autoimmune diseases such as Systemic lupus erythematosus (SLE) and systemic vasculitis (25). It is also now well accepted that neutrophils have an important role in the initiation and progression of cancer (5). Neutrophils’ involvement in a wide range of clinical conditions highlights the possibility that targeting neutrophils to modify their function may serve as a novel mode of immunotherapy in various inflammatory and autoimmune diseases as well as in cancer (26–29). While this may be true, their characteristic short life span makes *ex vivo* manipulation impractical for therapeutic purposes. Instead, neutrophil manipulation may be achieved *via* cell specific drug delivery *in vivo* (26).

In the current study we used a phage display library to identify short peptide sequences which specifically bind either murine or human neutrophils. We show that the identified peptides are benign and do not affect neutrophil activation or viability. Importantly, decorating nanoparticles with neutrophil-specific peptide confers neutrophil specificity and, depending on the payload, neutrophil-specific nanoparticles can modify various neutrophil functions both *in vitro* and *in vivo*. Furthermore, we demonstrate that when administered systemically, neutrophil-specific nanoparticles are taken up by circulating neutrophils that carry them to sites of inflammation. The ability to specifically modify neutrophil function enables the possibility of modulating neutrophil toxicity for a wide range of diseases.

## Results

Neutrophils are known to play critical roles in a wide range of pathologies. While possessing beneficial properties, under certain conditions their function may be harmful. In the case of COPD as an example, neutrophils were shown to generate ROS and release destructive proteases which destroy the lung parenchyma ultimately leading to emphysema (30–32). This suggests that targeting deleterious processes in neutrophils may be therapeutically beneficial. While this may be true, their characteristic short life span makes *ex vivo* manipulation impractical for therapeutic purposes (33). Instead, neutrophil manipulation may be achieved *via* cell specific drug delivery *in vivo* (26).

To design neutrophil-targeting molecules we hypothesized that relatively short peptides may specifically bind neutrophils yet would not yield neutrophil activation & depletion as caused by antibodies. To test this possibility, we used phage display to screen through 10^10^ 6-12aa long random peptide sequences (34). The screen was conducted using murine Normal-Density Neutrophils (NDN (19)) for positive selection whereas monocytes and lymphocytes served for negative selection (**Figure S1A**). Following 3 rounds of positive panning and 2 interlaced rounds of negative panning, the neutrophil affinity selected phages were eluted and sequenced (**Figure S1A**). Of the resulting sequences (**Figure S1B**) the sequence LQIQSWSSSP stood out as it was repeatedly isolated, constituting 28.4% of all phages enriched during the panning (**Figure S1C**). The phage presenting the LQIQSWSSSP peptide (designated LQI-peptide) continued to show the highest specificity, binding to 95% of mouse neutrophils, with no detectable binding to monocytes or lymphocytes (**Figures S1D, E** **)**. Interestingly, while the unlabeled LQI-peptide (**Figure S1F**) efficiently competed with LQI-phage binding to neutrophils, any modification of the peptide dramatically reduced its binding to neutrophils (**Figure S1G**).

**Figure 1:**
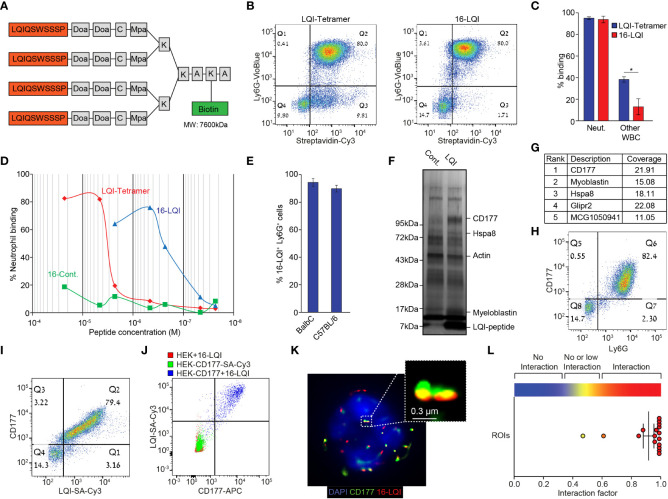
Murine Neutrophil-Specific LQI-Peptide binding *via* CD177. **(A)** Schematic representation of the biotin-LQI-tetramer. **(B)** FACS analysis comparing the binding of LQI tetramer (left) and 16-LQI (right) to WBC following 20 min. incubation with 1μM of each. **(C)** Quantification of LQI tetramer (blue) or 16-LQI (red) binding to neutrophils and other WBC. **(D)** Mouse neutrophils were incubated with the LQI-tetramer, the 16-LQI complex and the 16-Cont. (human neutrophil binding peptide - KFP) complex in increasing concentration. The samples were then analyzed by flow cytometry and the presented shows the fraction of Cy3^+^ neutrophils (Ly6G^+^). The 16-LQi and the 16-Cont. were preincubated with SA-Cy3, the LQI-tetramer was first incubated with the cells and then incubated with SA-Cy3. **(E)** Quantification of FACS analysis of the extent of binding of 16-LQI (1μM) to circulating Ly6G^+^ neutrophils from healthy Balb/C and C57BL/6 mice (n=3). **(F)** Silver staining of the gel plotting the proteins pulled down using naked (Cont.) and LQI-tetramer decorated (LQI-peptide) SA agarose beads. **(G)** Top ranking proteins enriched by LQI-tetramer-SA pull down. **(H, I)** Representative dot plots of isolated WBC stained with Ly6G and CD177 **(H)** and CD177 and 1μM LQI-tetramer **(I)**. **(J)** FACS analysis of 16-LQI binding to control HEK293T cells (Red), SA-Cy3 binding to HEK293T cells overexpressing the murine CD177 protein (Green) and CD177-overexpressing HEK293T cells were incubated with 16-LQI (blue). **(K)** Representative STORM imaging of a single neutrophil with staining of CD177 (green) and 16-LQI (red), overlay with brightfield (left) or DAPI staining of nucleus (right). **(L)** Quantification of CD177 and 16-LQI interaction for image depicted in **(K)** using the ImageJ Interaction Factor plugin (35). Error bars represent ± SEM. * p<0.05.

We noticed that fluorescently labeled free LQI peptide generated a low neutrophil binding signal and therefore generated a tetramer molecule where 4 repetitive LQI-peptide sequences are connected *via* a Doa-Mpa linker to a single biotin molecule (**Figure 1A**). When incubated with white blood cells (using Cy3-labeled streptavidin as a fluorophore) the LQI-tetramer efficiently binds nearly 100% of neutrophils, however it concomitantly binds ~50% of other WBC (**Figure 1B** left, **1C**). In contrast preincubation of the LQI-tetramer with Cy3-labeled streptavidin generates a tetramer of multiantigen molecules (total of 16 LQI peptide repeats, designated 16-LQI, **Figure 1B** right). The 16-LQI, showed 100% binding of neutrophils with only ~10% binding to other WBC (**Figures 1B, C** **)**. Further, the affinity of 16-LQI to neutrophils is ~100 times higher than that of the LQI-tetramer alone (**Figure 1D**). Next, we found that 16-LQI efficiently binds circulating neutrophils from both healthy Balb/c and C57Bl6 mice (**Figure 1E**). Finally, confocal imaging of cells incubated with 16-LQI shows it interacts specifically with murine neutrophils (**Figure S1H**).

Importantly, the binding of the LQI-tetramer to neutrophils has no significant effect on viability, activation or on ROS production (**Figures S2A-E**). In addition, the LQI-tetramer does not act as a chemoattractant (**Figure S2F**) and does not interfere with neutrophil chemoattraction (**Figure S2G**). Taken together, these observations suggest that the LQI-tetramer efficiently binds mouse neutrophils and does not interfere with neutrophil function.

Next, we investigated the binding target of the LQI peptide on the neutrophil surface. Using the biotinylated LQI-tetramer to decorate streptavidin agarose beads we captured interacting proteins from a murine neutrophil lysate. The enriched proteins were run on a gel and were visualized using silver staining (**Figure 1F**) prior to mass spectrometry. The highest-scoring hit in the mass spectrometry analysis was CD177, a neutrophil specific membrane associated protein (**Figure 1G**). Flow cytometry showed that >99% of Ly6G^+^ circulating murine neutrophils are also CD177^+^ whereas Ly6G^-^ cells are CD177^-^ (**Figure 1H**). Importantly, all circulating CD177^+^ cells (neutrophils) bind 16-LQI (**Figure 1I**). The fact that the LQI-peptide binds only to HEK293T cells ectopically expressing the murine CD177 confirms that CD177 is both required and sufficient for LQI-peptide binding (**Figure 1J**). Finally, using Stochastic Optical Reconstruction Microscopy (STORM) we found that the CD177 and 16-LQI signals overlap in >90% of the clusters, indicating that these molecules are in very close interaction (**Figures 1K, L** **)**.

**Figure 2:**
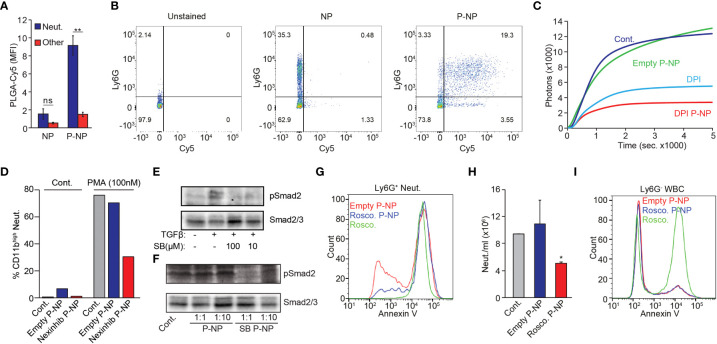
P-NP Mediated Modulation of Neutrophil Function *In Vitro.* **(A)** Representative quantification of uncoated or LQI-coated NP binding to neutrophils (blue) or other WBC (red). **(B)** FACS plots showing uncoated (middle) or LQI tetramer coated (right) Cy5-labelled PLGA NP binding to Ly6G^+^ neutrophils in whole blood. Left panel shows unstained control. RBC were lysed prior to FACS analysis. Cells with high side scatter (SSC) represent neutrophils. **(C)** PMA (50 nM) induced ROS production in control neutrophils (Cont.), neutrophils treated with empty (Empty NP) or DPI-containing P-NP (DPI NP) or with free DPI (DPI, 10 µM). **(D)** FACS analysis of CD11b expression in neutrophils before (Cont.) or following PMA (100 nM) stimulation. Neutrophils were preincubated with vehicle, empty or Nexinhib-20 loaded LQI tetramer coated NP^#^. The experiment was done several times in different combination of experimental conditions which cannot be averaged and presented with error bars. **(E)** Upper panel - western blot analysis showing Smad2 phosphorylation in neutrophils stimulated with TGFβ (10 ng/µl, 30 min.) in the presence of SB431542 (100 μM or 10 μM). Control neutrophils (leftmost lane) were treated with vehicle. Lower panel - western blot analysis of Smad 2/3 serving as loading control for the samples in the upper panel. **(F)** Western blot analysis of Smad2 phosphorylation and Smad2/3 expression in neutrophils which were pre-incubated with 6 or 0.6 μg/μl empty (1:10) NP, 6 or 0.6 μg/μl (1:10) SB431542 NP before stimulating with TGFβ (10 ng/μl, 30 min.). Control cells were treated only with TGFβ. Lower panel - western blot analysis of Smad 2/3 serving as loading control for the samples in the upper panel. **(G)** Ly6G^+^ Neutrophils isolated from 4T1-tumor bearing mice were incubated with empty P-NP, roscovitine-loaded P-NP or free Roscovitine. Subsequently cells were analyzed for Annexin-V binding to quantify apoptotic cells. **(H)** Effect of empty or roscovitine-containing P-NP on neutrophil numbers 4 hrs following P-NP administration. **(I)** Non-neutrophil Ly6G^-^ cells isolated from 4T1-tumor bearing mice were incubated with empty P-NP, roscovitine-loaded P-NP or free Roscovitine. Subsequently cells were analyzed for Annexin-V binding to quantify apoptotic cells. The experiments were repeated at least 3 times with similar results. Error bars represent ± SEM. * p<0.05, ** p<0.01. ns, not significant. ^#^The experiment was done several times in different combination of experimental conditions which cannot be averaged and presented with error bars.

To achieve neutrophil specific drug delivery, we next decorated nanoparticles with the LQI-tetramer to render them neutrophil-specific. We fabricated PLGA (poly(lactic-co-glycolic acid)) nanoparticles (NP) containing PLGA-PEG-Maleimide (30%) and designed an LQI-tetramer bearing a c-terminal cysteine instead of biotin. Incorporating PLGA-Cy5 (10%) in the NP formulation facilitated detection of NP uptake by flow cytometry. Following extensive optimization, we determined that the most efficient NP peptide conjugation for neutrophil targeting is *via* PLGA-PEG-Mal providing very high neutrophil uptake with minimal uptake by non-neutrophil WBC (**Figures S3A-E** **)**. Importantly, using electron microscopy we observed no size difference between peptide-coated (P-NP) and non-peptide coated NP (**Figure S3F**). Indeed, decorating NP with the peptide (P-NP) conferred neutrophil specificity and dramatically increases the uptake by neutrophils (**Figures 2A, B** **)**. Using 3D reconstitution of confocal imaging, we demonstrated that the P-NP are taken up by neutrophils (**Movie S1**).

PLGA nanoparticles are known to gradually degrade within the cell, releasing encapsulated payload (36). We therefore tested whether neutrophil degranulation or ROS production could be modulated by encapsulating small molecule inhibitors within neutrophil specific P-NP. Neutrophils generate ROS *via* the NADPH oxidase complex which is potently inhibited by Diphenyleneiodonium (DPI). Stimulating neutrophils with Phorbol 12-myristate 13-acetate (PMA) dramatically increases ROS production (**Figure 2C**). While addition of empty P-NP had no effect on PMA stimulated ROS production, free DPI, and to a greater extent DPI-containing P-NP, efficiently blocked the effect of PMA on ROS production (**Figure 2C**). Using a similar strategy, we tested whether P-NP may be used to block neutrophils’ ability to degranulate, a process that may be inhibited by Nexinhib-20 (37). Following PMA stimulation neutrophils degranulate, as shown by an increase in CD11b surface expression. This process is dramatically lowered when neutrophils are pre-treated with Nexinhib-20 containing P-NP (**Figure 2D**). We next tested whether neutrophil-specific targeting may be used to modify intracellular signaling. To the end we used SB431542, a small molecule inhibitor of TGFβ signaling loaded into P-NP. Notably, while neutrophils stimulated with TGFβ show an increase in Smad2 phosphorylation, in the presence of SB431542 Smad2 phosphorylation is blocked (**Figure 2E**). In a similar fashion, while empty P-NP have no effect on TGFβ stimulated neutrophil Smad2 phosphorylation, SB431542-containing P-NP efficiently down-regulate the phosphorylation of Smad2 (**Figure 2F**). Finally, we tested whether neutrophil viability may be manipulated by specific targeting of Roscovitine. Our data show that in comparison with empty P-NP, Roscovitine containing P-NP increase the fraction of Ly6G^+^ neutrophils undergoing apoptosis, reaching levels similar to those of free Roscovitine (**Figures 2G, H**). Importantly, while free Roscovitine dramatically increases the rate of apoptosis in non-neutrophil Ly6G- cells, encapsulated Roscovitine has no effect of their rate of apoptosis (compared with empty P-NP, **Figure 2I**). This suggests that encapsulating Roscovitine within P-NP can be used to induce apoptosis in a neutrophil specific manner.

**Figure 3:**
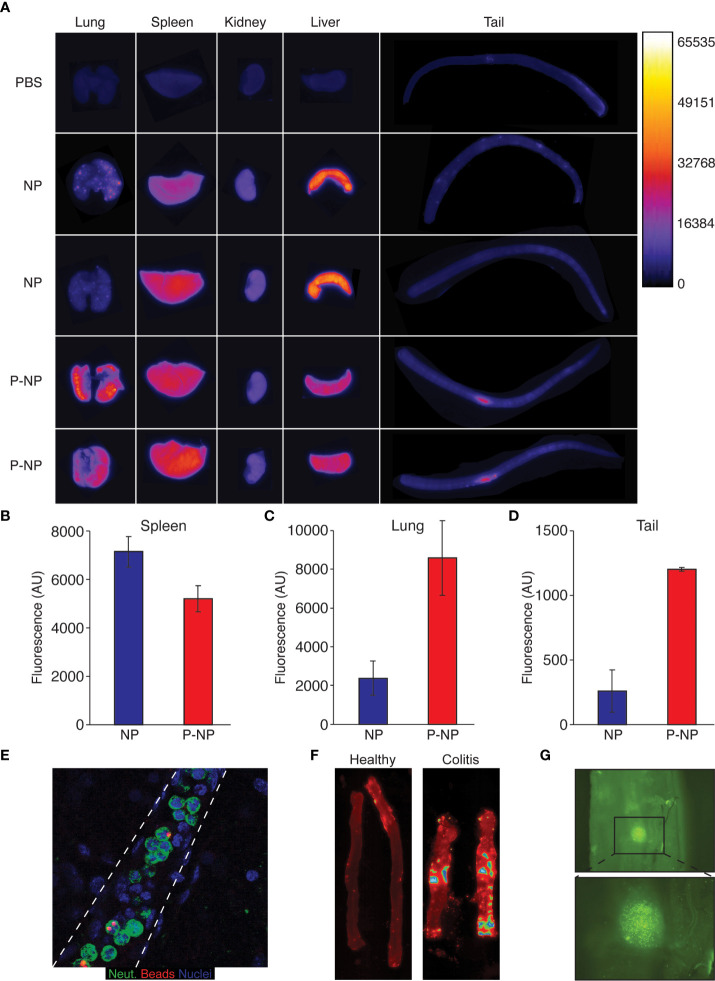
Nanoparticle distribution *in vivo.* For biodistribution experiments we used Cy3^+^-streptavidin nanobeads which were either administered as naked nanobeads (NB) or following labelling with the LQI neutrophil targeting peptide (P-NB). **(A)** Typhoon™ biodistribution imaging in healthy Balb/C mice 3 hours following *i.v.* injection of either Cy3^+^ NB, Cy3^+^ P-NB or PBS as control. The mice were sacrificed and their organs imaged immediately. **(B-D)** Quantification of Typhoon Cy3 fluorescence reading from the spleen **(B)**, the lungs **(C)** and the tail **(D)** of mice injected with either NB or P-NB/**(E)** Fluorescent staining of a neutrophils (green) carrying fluorescent nanobeads (red) within a blood vessel (dashed lines). **(F)** Representative Typhoon™ imaging of Cy3^+^ P-NB in colons from healthy mice (left) and mice with DSS-induced colitis (right). The mice were sacrificed 3 hours following *i.v.* injection Cy3^+^ P-NB, the colon harvested, washed with PBS and imaged immediately. **(G)** Representative imaging taken with a fluorescent binocular showing the accumulation of P-NB the inflamed colon from mouse with DSS-induced colitis. Following the induction of colitis, the mouse was injected with Cy3^+^ P-NB, the colon harvested, washed with PBS and imaged immediately. Error bars represent ± SEM.

Together, these observations demonstrate both the efficacy and specificity of using P-NP to specifically manipulate processes within neutrophils that are critical in the context of inflammation. We then analyzed the biodistribution of Cy3^+^ streptavidin-coated nanobeads (NB) using Typhoon™ (38) and found that splenic accumulation of NB was very high but was lower when using LQI-peptide decorated NB (P-NB) (**Figures 3A, B** **)**. In contrast, P-NB accumulation in the lungs was significantly higher than that of NB (**Figures 3A, C** **)** reflecting the presence of lung resident neutrophils. We also noticed that the site of *i.v.* injection in the tail shows accumulation of P-NB but not of uncoated NB (**Figures 3A, D, E** **)** suggesting that P-NB are taken up by circulating neutrophils which are recruited to the site of inflammation caused by injection.

To further examine nanoparticle labeled neutrophil recruitment, we used the DSS-induced mouse model of colitis. Healthy and colitic mice were injected *i.v.* with P-NB and Cy3 fluorescence in their colons were evaluated using Typhoon™. While there is no fluorescence emanating from the colons of healthy mice (left panel), there are foci within the inflamed colon (right panel) with very high Cy3 signal representing the accumulation of P-NB (**Figure 3F** **)**. Indeed, observing the inflamed colon using fluorescent imaging (4X) shows the accumulation of clusters of Cy3^+^ cells in this site (**Figure 3G**).

**Figure 4:**
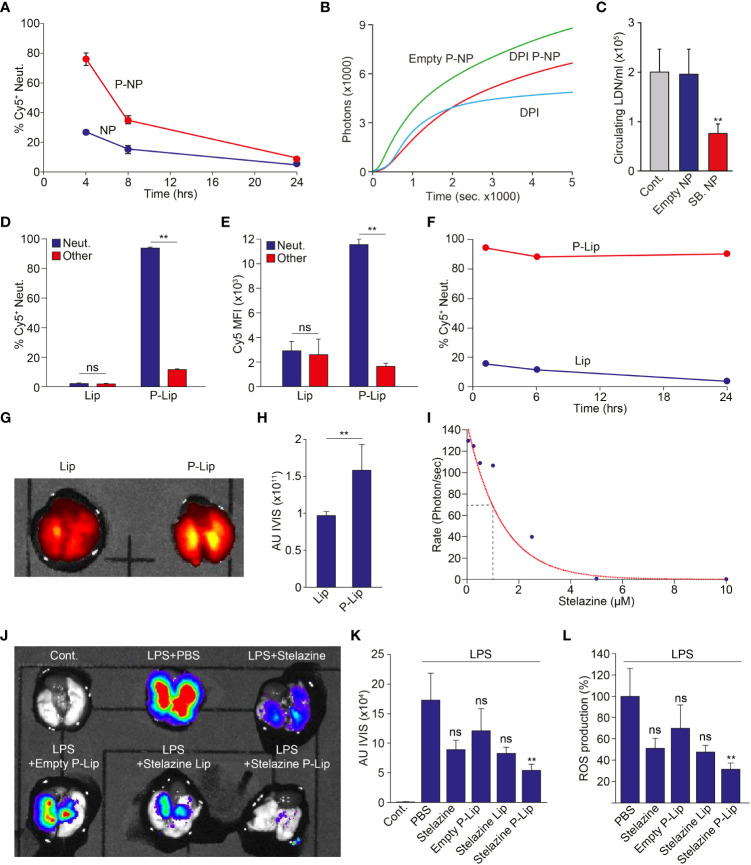
Modulation of Neutrophil Function *In Vivo.* **(A)** NP containing neutrophils in healthy Balb/C mice injected *i.v.* with either NP or P-NP at 4, 8 and 24 hours post injection (n=2 per group). **(B)** Healthy Balb/c mice were intraperitoneally injected with Zymosan-A to induce peritonitis. These mice were then i.v. injected with free DPI, empty P-NP or DPI containing P-NP. The peritoneal neutrophils were then isolated and stimulated with PMA (100 nM). Graph shows the extent of H_2_O_2_ production in neutrophils isolated from treated mice following PMA stimulation. **(C)** SB431542-containing P-NP limit the propagation of circulating low density neutrophils in 4T1 tumor bearing mice, indicating effective blocking of TGFβ *in vivo* (19). Control mice (Cont.) were untreated and mice treated with empty P-NP (Empty NP) served as control for P-NP injection. **(D)** The extent of targeting Cy5^+^ Lip and P-to neutrophils (blue) or other WBCs (red) 4 hrs post *i.v.* administration (n=4 per group). **(E)** The relative uptake (Cy5 MFI) of Cy5^+^ Lip and Cy5^+^ P-Lip by neutrophils (blue) or other WBCs (red) 4 hrs post *i.v.* administration (n=4 per group). **(F)** Lip-containing neutrophils in healthy Balb/C mice injected *i.v.* with either Lip or P-Lip at 1, 6 and 24 hours post injection (n=4 per group). **(G)** Representative imaging of Cy5 emanating from the lungs of mice with ARDS treated with Cy5^+^ Lip or Cy5^+^ P-Lip. **(H)** Average intensity of Cy5 emanating from the lungs of mice with ARDS treated with Cy5^+^ Lip or Cy5^+^ P-Lip (n=4 per group). **(I)** Stelazine dose dependent inhibition of ROS production in isolated neutrophils. **(J)** Representative IVIS imaging of ROS production emanating from lungs extracted from a control mouse and mice treated with LPS. LPS treated mice were further treated with PBS, free Stelazine, empty P-Lip, Stelazine Lip and Stelazine P-Lip. **(K)** Average IVIS reading from control mice and LPS treated mice that were also treated with PBS, free Stelazine, empty P-Lip, Stelazine Lip and Stelazine P-Lip (n=4 per group). **(L)** Average ROS production in LPS treated mice that were also treated with PBS, free Stelazine, empty P-Lip, Stelazine Lip and Stelazine P-Lip. LPS treated mice treated with PBS were used as 100% (n=4 per group). The experiments were repeated at least 3 times with similar results. Error bars represent ± SEM. ** p<0.01. ns, not significant.

Finally, we investigated whether P-NP may be used to modify neutrophil function *in vivo*. First, we injected P-NP *i.v.* to healthy mice and monitored particle uptake and distribution. We found that 4 hrs following *i.v.* administration, ~80% of circulating neutrophils are labeled by P-NP whereas fewer than 30% of circulating neutrophils are labeled by NP (**Figure 4A**). However, the extent of neutrophil association with these PLGA P-NP rapidly decreases with a half-life of approximately 4 hrs (**Figure 4A**). Still, we tested whether P-NP loaded with specific neutrophil inhibitory small molecules can modify neutrophil function *in vivo*. We found that while empty P-NP had no effect on ROS production, P-NP containing DPI administered in a mouse model of peritonitis, significantly reduced ROS production (**Figure 4B**). Notably, blocking of ROS production by DPI containing P-NP was similar to the blocking achieved using free DPI suggesting that targeting DPI specifically to neutrophils is as efficient as systemic DPI administration and may serve as a mode for circumventing the toxicity associated with systemic administration of DPI. In a previous study we demonstrated that neutrophils exist in two phenotypically distinct subsets, normal-density (NDN) and low-density (LDN (19)). We further showed that TGFβ drives the transition of NDN to the LDN and that blocking of TGFβ reduces the numbers of circulating LDN. Here we show that in a similar fashion, while empty P-NP had little effect on LDN numbers in the circulation of 4T1 tumor bearing mice, SB431542-containing P-NP administered *i.v.* dramatically reduced LDN numbers (**Figure 4C**).

The relatively short half-life of the neutrophil targeting PLGA nanoparticles (~4 hrs, **Figure 4A**) represents a major challenge for application of these molecules in the clinic. We therefore explored the possibility of using liposomes, which have previously demonstrated extended nanoparticle pharmacokinetics to prolong the duration of effective neutrophil targeting. To this end we fabricated either standard liposomes (Lip) or peptide decorated liposomes (P-Lip). As seen with the PLGA NP, decorating liposomes dramatically enhances both the fraction of targeted neutrophils (% Cy5^+^ neutrophils, **Figure 4D**) and the number of liposomes per cell (average Cy5 MFI, **Figure 4E**). These observations show that decorating liposomes with the neutrophil-specific peptide enhances both the specific delivery to neutrophils and the number of liposomes per neutrophil. Strikingly, we also noted that the duration of P-Lip labeling of neutrophils is dramatically increased (**Figure 4F**) when compared with P-NP.

Next, we used a mouse model of Acute Respiratory Distress Syndrome (ARDS) and noted that neutrophil targeted liposomes (P-Lip, **Figures 4G, H** **)** accumulate in the inflamed lungs significantly more than non targeted liposomes (Lip, **Figures 4G, H** **)**. We then tested the efficacy of neutrophil targeted liposomal drug delivery on neutrophil function in the ARDS model. This again presented a difficulty since liposomes consist of a hydrophilic lumen which is incompatible with the neutrophil modulating small molecules used thus far (e.g., DPI). We therefore performed a high throughput screen for hydrophilic drugs that efficiently block neutrophil ROS production and found that Stelazine, an anti-psychotic drug, efficiently blocks neutrophil ROS production of activated neutrophils with an IC50 of ~ 1μM (**Figure 4I**). We then tested whether targeting Stelazine to neutrophils using P-Lip can block ROS production *in vivo*. We compared the extent of ROS production in LPS induced ARDS while treating the mice with vehicle, free Stelazine, Stelazine in non-targeted liposomes (Stelazine-Lip) and Stelazine in targeted liposomes (Stelazine P-Lip). While free Stelazine and Stelazine-Lip had some effect on neutrophil ROS production, targeted delivery of Stelazine to neutrophils in Stelazine P-Lip reduced neutrophil ROS production by >65% (**Figures 4J-L**).

**Figure 5:**
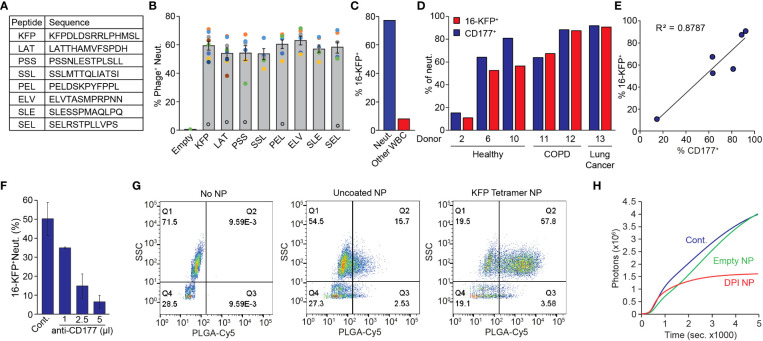
Characterization of Human P-NP. **(A)** High ranking human neutrophil binding peptide sequences. **(B)** Binding of phages expressing different peptides to CD66b^+^ neutrophils from different donors (colored circles). Open black circles represent an outlier excluded from the overall average. **(C)** Representative quantification of 16-KFP binding to neutrophils (SSC^high^, blue) and other WBC (SSC^low^, red) from a healthy donor. **(D)** 16-KFP binding and CD177 expression of neutrophils of healthy donors (2, 6, 10), COPD patients (11, 12) and lung cancer patients (13). Note the low neutrophil binding and low CD177 expression in healthy donor #2. **(E)** Correlation between CD177 expression and 16-KFP binding in different donors. **(F)** Binding of 16-KFP to neutrophils with increasing amounts of CD177 blocking antibody. **(G)** FACS analysis of human neutrophil binding of KFP-tetramer coated (right) and uncoated (middle) PLGA-Cy5 labelled NP in WBC from a healthy donor. **(H)** PMA induced ROS production in control neutrophils (Cont.), neutrophils treated with empty (Empty NP) or DPI-containing P-NP (DPI NP).

Using a strategy similar to the one described above (**Figure S1** **)**, we screened our phage display library for peptides specifically binding human neutrophils. This strategy yielded 8 different peptides (**Figure 5A**) which specifically bind human neutrophils when presented on the phage surface. Notably, phages expressing the various neutrophil binding peptides all bound ~60% of circulating neutrophils obtained from healthy donors (**Figure 5B**) suggesting a common binding mechanism. Nonetheless, these phages consistently bound ~5% of neutrophils from one specific healthy donor (black empty circles, **Figure 5B**) suggesting that the binding target is lacking in this individual. We focused on one peptide (KFPDLDSRRLPHMSL - KFP) and generated a biotinylated KFP-tetramer multiantigen (designated 16-KFP) for downstream applications. In whole blood, 16-KFP bound ~80% of human neutrophils and less than 10% of non-neutrophil WBC (**Figure 5C**). The human 16-KFP has no significant effect on neutrophil activation, viability or chemotactic properties (**Figure S4**). Using the KFP-tetramer as bait in a pull-down experiment followed by mass spectrometry, we again identified CD177 the binding partner for 16-KFP. Indeed, although the number of samples is limited, we found a correlation between CD177 expression and KFP-tetramer binding to neutrophils of healthy donors, patients with COPD and lung cancer patients (**Figures 5D, E**; **Table S1**). Importantly, we found that healthy donor #2, whose neutrophils were bound only to a low percentage by the neutrophil-specific phages (**Figure 5D**), is in fact a CD177 hypomorph (as are ~5% of the population (39)) (**Figure 5D**). The low binding of KFP to the neutrophils from donor #2 (of which only 5% express its binding partner CD177) supports the notion that KFP binding to neutrophils requires CD177 expression. Moreover, the extent of KFP-tetramer neutrophil binding and CD177 levels fit well within the correlation plot and support the role of CD177 in mediating the binding of the KFP-tetramer to neutrophils (**Figure 5E**). Further corroborating the role of CD177 in this process, we demonstrated that increasing concentrations of a human CD177 antibody effectively compete with the binding of the 16-KFP (**Figure 5F**). Intriguingly, the mouse and human CD177 proteins act as binding partners for our neutrophil specific peptides though they differ in molecular weight [105kDa and 54kDa respectively (40)] and the 16-LQI and the 16-KFP do not cross bind (see **Figure 1D**, 16-Cont.).

Next, we tested whether human P-NP may be used to modify human neutrophil function *in vitro*. Coating the PLGA/PLGA-PEG-Maleimide NP with the KFP-tetramer indeed confers neutrophil specificity (**Figure 5G**). We then demonstrated that DPI-loaded, but not empty P-NP, efficiently block PMA-induced ROS production in human neutrophils (**Figure 5H**).

## Discussion

Neutrophils are the most abundant white blood cell population in the circulation. Although previously thought to have a limited functional plasticity this view has changed due to findings made in recent years. Neutrophils were found to play various, sometimes conflicting, roles in many clinical scenarios ranging from periodontal diseases to cancer (7). This new understanding has made it clear that neutrophils, being numerous and highly active in a variety of diseases, may serve as a therapeutic target for therapeutic treatment. However, this was considered difficult due to the unique nature of neutrophils. Neutrophils are short lived (2-5 days at the most in the human circulation) and easily activated yielding cell death. This precludes the option of harvesting and modifying neutrophils for therapeutic purposes the same way T cells are currently modified in CAR-T. Instead, neutrophils must be modified *in vivo*, by targeted molecules with an extended half-life to account for constant turnover (26). Indeed, there are ongoing efforts to modify neutrophils therapeutically *via* systemic blocking of neutrophil recruitment, mobilization or activation. However, there are no FDA approved neutrophil targeting molecules and those that proceeded through clinical trials showed significant side effects, including increased infections which may be a result of neutropenia (41, 42).

With this realization we postulated that neutrophil targeted drug delivery, aimed at blocking specific neutrophil functions, will circumvent several hurdles associated with utilizing neutrophils for therapeutic purposes: 1) It will allow modification of neutrophils *in vivo*. 2) It will allow the use of potentially toxic compounds that are not tolerated well when administered systemically. 3) It will block specific neutrophil functions without eliminating neutrophils altogether. To this end we used a phage display library to screen for short peptides which bind neutrophils in a specific fashion. In the mouse phage display screen, we identified a single neutrophil specific peptide. It was highly represented and bound neutrophils with increased specificity when expressed in multiple copies on the phage surface (**Figure 1**). With the requirement for both high affinity and high avidity, achieving similar binding of the purified peptide required a unique chemical structure where 4 peptide repeats are linked together on a single peptide backbone. Importantly, this peptide had no apparent effect on neutrophil function or viability. When used to decorate PLGA nanoparticles or liposomes the neutrophil-specific peptide confers neutrophil targeting specificity and was found to bind all subsets of neutrophils including NDN, LDN, inflammatory neutrophils and tumor associated neutrophils (TAN, data not shown). PLGA nanoparticles and liposomes decorated with the peptide not only bind neutrophils specifically *in vitro* but also *in vivo*. Furthermore, they are taken up by neutrophils *in vivo* and are carried to the sites of inflammation where they accumulate.

We also showed that we can effectively use drugs loaded nanoparticles to modulate neutrophil activity by blocking the production of Reactive Oxygen Species (ROS), neutrophil degranulation, TGFβ signaling and even induce apoptosis in a specific manner (**Figure 2**). Lastly, we were able to identify human neutrophil binding peptides which, like the mouse peptide, provide neutrophil specificity to nanoparticles when they are presented on the particle surface. Taken together, these observations suggest that targeted nanoparticles (PLGA or liposomes) may be used as a platform for modifying specific neutrophil functions *in vivo* without inhibiting other important functions of these cells.

Neutrophils play a key role in a wide array of clinical conditions including inflammatory diseases, severe reactions to infections (e.g., sepsis or ARDS) and cancer. Neutrophil targeted nanoparticles may be used to limit inflammation (ROS, degranulation, chemotaxis), enhance anti-tumor immunity (TGFβ), decrease NETosis (PAD4) and more. In this respect, neutrophil specific drug delivery may be viewed as a platform that may be custom tailored to benefit patients in a broad spectrum of clinical conditions. These findings create a new avenue in immunotherapy by establishing a platform delivery modality to modulate neutrophils.

## Materials and methods

### Animals

5-6-week-old BALB/c and C57BL/6 mice were purchased from Harlan (Israel). All experiments involving animals were approved by the Hebrew University’s Institutional Animal Care and Use Committee (IACUC).

### Cell lines

4T1 and HEK293T cells were purchased from the ATCC. HEK293T cells were infected with a lentiviral vector (pLVX-mCD177) to stably express the murine CD177. All cells were cultured in DMEM with 10% FCS, 2 mM L-glutamine and 100 U/ml penicillin/100 μg/ml streptomycin.

### Antibodies

Antibodies used in this study were the following: rat anti mouse Ly6G-violet 450 (Tonbo Biosciences), rat anti mouse Gr1-FITC (Tonbo Biosciences), rat anti mouse/human CD45-PE (BD Biosciences), rat anti mouse/human CD45-APC (Bio Legend), rat anti mouse/human CD11b-FITC (BD Biosciences), rat anti mouse CD177-Alexa Fluor 647 (R&D Systems), rat anti human CD177-APC (Bio Legend), mouse anti phage M13-PE (Santa Cruz), rabbit anti mouse/human pSmad2 (Cell Signaling), rabbit anti mouse/human pSmad3 (Abcam), rabbit anti mouse/human Smad2/3 (Cell Signaling), Annexin-V FITC (Biotium) and SA-Alexa Fluor 568 (Thermo Scientific).

### Peptides

Peptides used in this study were the following: LQIQSWSSSP and Cy5-LQIQSWSSSP (Sigma), Biotin-LQIQSWSSSP and Fluorescein-GGGS-RG-LQISWSSSP-AAG-CONH_2_ (by solid phase peptide synthesis (SPPS) as previously described by (43)). LQIQSWSSSP-Doa-Doa-C)_4_-(Mpa)_4_-Lys_2_-Lys-β-Ala-Lys(Biotin)-β-Ala-OH, (LQIQSWSSSP-Doa-Doa-C)_4_-(Mpa)_4_-Lys_2_-Lys-Cys-NH_2_, (KFPDLDSRRLPHMSL-Doa-Doa-C)_4_-(Mpa)_4_-Lys_2_-Lys-β-Ala-Lys(Biotin)-β-Ala-OH and (KFPDLDSRRLPHMSL-Doa-Doa-C)_4_-(Mpa)_4_-Lys_2_-Lys-Cys-NH_2_ (Intavis, Tübingen, Germany). The tetramer peptides were synthesized with a structure similar to multimeric peptides described by Li *et al.* in 2009 (44). The LQI tetramer presents the LQIQSWSSSP peptide on 4 branches with a free N-terminus connected by maleimide tetrameric cores. In addition to the peptide sequence two residues of 8-amino-3,6-dioxaoctanoic acid (Doa) were attached to the peptide to increase solubility. The C-terminus had either a biotin-tag which makes it accessible for fluorescent detection with Cy3-labeled streptavidin or a cysteine residue which allowed a click reaction to PLGA-PEG-Maleimide (structure in **Figure 1**). The human KFP tetramer was designed in the same fashion.

### Mouse models of disease

Cancer - Female 6-8 weeks old Balb/C mice were injected with 0.5x10^6^ of 4T1 tumor cells into the mammary fat pad or 1x10^6^ AB12 mesothelioma cells subcutaneously to the flank. Tumor growth was monitored and mice were sacrificed after 15-25 days depending on tumor size. The tumors were allowed to reach an average size of (700-800 mm^3^). Colitis - Colitis was induced in healthy male C57/Bl6 mice by adding 4% DSS to the drinking water for 5 consecutive days. The mice were then given DSS-free water for 2 days. ARDS - Healthy male C57/Bl6 mice were anesthetized with Xylazine/Ketamine and LPS (7.5mg/kg in 50ul PBS) was introduced by inhalation into the lungs. Control mice were treated with PBS. Measurement of ROS in the lungs was done using IVIS imaging at 48 hrs post LPS administration.

### Mouse neutrophil purification

Circulating Neutrophils were purified from orthotopically injected (mammary fat pad) 3-week 4T1 tumor-bearing mice as described before (45). In brief, whole blood was collected by cardiac puncture using heparinized (Sigma) syringe. The blood was diluted to a volume of 6 ml with 0.5% BSA/PBS and subjected to a discontinuous Histopaque (Sigma) gradient (1.077 and 1.119). NDN were collected from the 1.077-1.119 interface. LDN were collected from the plasma-1.077 interface. Red blood cells (RBCs) were eliminated by hypotonic lysis. Neutrophil purity and viability were determined visually and were consistently >98% for NDN. Tumor associated neutrophils were isolated from 4T1 tumors that were minced and digested in L15 medium (Sigma-Aldrich) containing 0.2 mg/mL collagenase type I, 0.05 mg/mL collagenase II, 0.2 mg/mL collagenase type IV (all from Sigma-Aldrich), 0.025 mg/mL DNase I (Roche Applied Science), and 0.025 mg/mL elastase at 37C for 40 minutes. The digested tissue mixture was then filtered through a 70-mm filter and centrifuged at 1,250 rpm, 5 minutes at room temperature. The pellet was then resuspended in RBC lysis buffer for 1 minute, RPMI medium was added to stop the lysis, and centrifuged again 1,250 rpm, 5 minutes at room temperature. Pellet was resuspended in EasySep buffer, and Ly6G^+^ cells were isolated using the EasySep APC-positive Selection Kit (STEMCELL Technologies) with anti-mouse Ly6G APC antibody (BioLegend) according to the manufacturer’s protocol.

### Human neutrophil purification

Collection of blood from patients was approved by Hadassah Medical Center institutional review board (IRB). Following informed consent, blood samples (~10cc) were collected from healthy volunteers or from lung or breast cancer patients. Blood samples were transferred to the lab for analysis no more than 15 minutes post-blood draw. Neutrophils were purified as previously described (45). In brief, heparinized blood (20U/ml) was mixed with an equal volume of Dextran 500 (3% in saline) and incubated 30 minutes at room temperature. The leukocyte-rich supernatant was layered on top of Histopaque 1077 (Sigma) and centrifuged. High-density neutrophils were collected in the pellet fraction. LDN were collected from the 1077-plasma interface. Neutrophils were resuspended in 10ml 0.2% NaCl for 30 seconds to remove contaminating erythrocytes. Isotonicity was restored by the addition of 10 ml 1.6% NaCl. Neutrophils were then washed three times in HBSS.

### Phage screening

Murine - The phage library stock (lib-8 mix) had a concentration of 1x10^12^ pfu/ml (34). The stock was diluted 1:10 in 0.5% BSA/1xPBS and 200 μl were incubated with 2x10^7^ purified NDN from 4T1 tumor bearing mice. The eluate was amplified in DH5αF^+^ E. coli and negatively selected on circulating lymphocytes and monocytes purified for healthy mice. Following 2 rounds of positive selection and an additional round of negative selection the phages were amplified by PCR (fwd – cgtcggcagcgtcagatgtgtataagagacagggccaacgtggc, rev – gtctcgtgggctcggagatgtgtataagagacagggccccagaccd) and subjected to deep sequencing by MiSeq (Core Research Facility Unit Hadassah Ein Kerem). The screen on murine NDN was conducted in 5 replicates in parallel. Human – Using a similar protocol phage panning was done on neutrophils isolated from 5 healthy donors (**Table S1**). The positive selections rounds were performed on NDN and the negative selection was performed using of mononuclear low-density cells. Final eluates were subjected to PCRs, purified and sequenced as described above.

### Neutrophil functional assays

ROS production - 2x10^5^ purified NDN in HBSS without phenol red were plated in white 96- flat-bottom well plate. 20 μl of a 500 μM luminol solution in HBSS including 40 U/ml HRP is added to each well. Then cells were treated with various amounts of the LQI tetramer peptide, 10 nM PMA or 100 nM PMA. Chemiluminescence was measured for 90 min (InfiniteF200Pro, TECAN) (46).

Migration - NDN were isolated by density-gradient centrifugation from the circulation of 4T1 tumor-bearing mice. 800 μl of 2% FCS/RPMI medium was placed into the 24-well plate, then Millicell^®^ cell culture inserts with a pore size of 5 μM were placed into the wells and were allowed to soak for 5 min. NDN were suspended to a density of 2.5x10^5^ cells/ml in 2% FCS/RPMI and 200 μl of the cell suspension was placed into the upper chamber of the transwell insert (46). Migration was stimulated by added 100 ng/μl CXCL2 to the bottom chamber of the wells (consider V=800 μl), and the LQI tetramer peptide was added to either in the lower chamber or to NDN in the upper chamber in different concentrations. The assay was stopped after 90 min. Each condition was tested in triplicate. Per well 5 pictures were taken at the same coordinates (middle, top, bottom, left, right). Cells per field of view were counted using ImageJ.

### Protein pulldown and mass spectrometry

Protein lysate (10^7^ NDN purified from 4T1-tumor bearing mice) were incubated for 3 hrs with 25 µl of LQI-SA-agarose beads (SA-agarose beads served as controls). Following washing, the beads were resuspended in 50 µl of 1.5x protein sample buffer (incl. 3% SDS and β-mercaptoethanol) and incubated for 15 min at 95°C. After short spin down, 35 µl of the supernatant were loaded on to a pre-cast gradient SDS-PAGE, the gel was stained with Pierce Silver Stain Kit according to manufacturer’s instructions. Relevant lanes were excised and sent for mass spectrometry analysis to the Smoler Proteomics Center (Technion, Haifa).

### Overexpression of murine CD177 in HEK293T cells

The murine CD177 generated a PCR using the primers AgeI-CD177 mouse and NheI-CD177 mouse (fwd – cagtacgaccggtgccgccaccatgaattctataccagtgctgaccc, rev –aaggctactagctagcgcagagaggacagatcccagcatac) on NDN cDNA. The mCD177 PCR product was digested with the restriction enzymes AgeI and NheI and cloned into the pLVX plasmid. HEK293T cells were infected with the generated lentiviral vector pLVX-mCD177. Overexpression of mCD177 was determined using anti-CD177-Alexa647 in flow cytometry analysis using parental HEK293T cells as control.

### Stochastic optical reconstruction microscopy imaging and analysis

STORM analysis was performed as previously described (47). In brief, WBC were isolated from 4T1-tumor bearing mice and incubated in 5 ml glycine quenching buffer for 30 min at RT. The cells were incubated with the LQI tetramer SA-Alexa 568 and CD177-Alexa 647 antibody for 30 min at 4°C. Following washing and fixation (PFA) the cells were stained with YOYO-1. Before imaging, cells were resuspended in STORM imaging buffer (100mM MEA, 10% glucose, Glox (11.2 mg/ml glucose oxidase (Sigma) and 1.8 mg/ml catalase (Sigma)) in dilution buffer (50 mM NaCl, 200 mM Tris in D_2_O)). STORM was performed in a Nikon Eclipse Ti-E microscope with a CFI Apo TIRF × 100 DIC N2 oil objective (NA 1.49, WD 0.12 mm) equipped with a Andor iXon-897 EMCCD camera. Super-resolution images were reconstructed from a series of at least 5000 images per channel using the N-STORM analysis module, version 1.1.21 of NIS Elements AR v. 4.40 (Laboratory imaging s.r.o.). Co-localization analysis was performed using the ImageJ plugin ‘Interaction Factor package’ described previously (35). The calculated ‘Interaction Factor’ scores between 0 and 1, where 0 represents no interaction and 1 represents complete overlap.

### Nanoparticle fabrication

PLGA - NP were synthesized by single emulsion method using PLGA (60%), PLGA-PEG-Maleimide (30%) and PLGA-Cy5 (10%) in the presence or absence of payload compound. For peptide coating, NP were incubated overnight at 4°C. The NP were washed, resuspended in PBS and loaded onto a dialysis tube with a cut-off of 300 kDa. Following dialysis, the NP were washed, suspended in PBS stored at 4°C in the dark until use. NP were measured using dynamic light scattering (Zetasizer Nano-ZSP, Malvern Instruments, UK). NP morphology was evaluated by means of transmission electron microscopy (TEM, Hebrew University core facility). To quantify drug loading, NP were dissolved in DMSO, sonicated and analyzed for absorbance at the wavelength specific to the drug (SB-431542 = 325 nm, DPI = 257 nm, roscovitine = 293 nm, Nexinhib20 = 272 nm). NP were fabricated using: PLGA (50:50), acid terminated Mw 38,000-54,000 (Resomer^®^ RG 504 H, acid terminated, Sigma); PLGA-Cy5 (50:50) Mw 45,000 – 55,000 (AV034, PolySciTech^®^, Akina, Inc.), PLGA(50:50) (30,000)-PEG(5,000)-Maleimide Mw ~30,000-55,000 (AI110, PolySciTech^®^, Akina, Inc.).

Streptavidin Coated Fluorescent Nile Red Particles (0.7-0.9 μM) were purchased from Spherotech, Inc., IL, US. The nanospheres were decorated with the LQI-tetramer using Streptavidin-Biotin interactions.

Liposomes – Liposomes were fabricated using HSPC (Lipoid), Cholesterol (Sigma), DSPE-PEG3400-Mal (NOF) and DilC18 (5)-DS at a molar ratio of 56.5: 41: 2.5: 0.18. Liposomes were fabricated as described before (48, 49). The liposomes were incubated with the targeting peptide for 1.5hr at RT on shaker (20 rpm), and then incubated overnight at 4°C on shaker (20 rpm). Free maleimide was quenched using 1mM L-Cysteine. Small molecule drugs were actively loaded into liposomes which were then cleansed using PD-10 columns.

### High throughput screening

High throughput screening for molecules blocking neutrophil ROS was done at the INCPM (Weizmann Institute, Israel). 2000 human neutrophils were plated onto 384 plates containing 10µM compounds from the Sigma LOPAC collection, Prestwick Known Drugs, Selleck Chemicals Screening collections (there are many subsets there), Targetmol FDA approved cancer and the Microsource Spectrum Collection. Following 30 min. incubation the neutrophils were stimulated PMA and the production of ROS was measured as described above. Positive hits which were found to affect neutrophil viability or the ROS production assay were discarded.

### *In vivo* NP administration

Biodistribution - Healthy 6-8 weeks old Balb/C mice were injected *i.v.* with 30 μl of Cy5-labeled NP or P-NP (in 200 μl PBS). 3 hours later mice were sacrificed, organs were extracted and imaged immediately using the Typhoon™ FLA 7000 biomolecular Imager as previously described (38). NP were similarly administered to 7-8 weeks old male C57Bl/6 mice with DSS-induced colitis.

Roscovitine-loaded NP - 6-8 week old male Balb/C mice were injected *i.v.* with 200 μl empty or roscovitine-containing P-NP in sterile PBS (5 μg). Control mice were treated with vehicle. 4 hours after NP administration mice were sacrificed, blood was extracted by cardiac puncture and neutrophils were quantified by flow cytometry.>

DPI- and Nexinhib20-loaded NP - Mice were injected *i.v.* with 200 μl of empty, DPI-containing P-NP (1.2µg/mouse) or Nexinhib20-containing P-NP (1.56µg/mouse). Stelazine – Mice were injected *i.v.* with 200 μl of empty P-NP, Stelazine-containing NP or Stelazine-containing P-NP (6.6mg/kg for both).

### Statistical analysis

For studies comparing differences between two groups, we used unpaired Student’s t tests. For studies comparing more than two groups, we used ANOVA with appropriate *post hoc* testing. Differences were considered significant when p < 0.05. Data are presented as mean ± SEM.

## Data availability statement

The original contributions presented in the study are included in the article/**Supplementary Material**. Further inquiries can be directed to the corresponding authors.

## Ethics statement

The studies involving human participants were reviewed and approved by Hadassah Medical Center institutional review board (IRB). The patients/participants provided their written informed consent to participate in this study. All experiments involving animals were approved by the Hebrew University’s Institutional Animal Care and Use Committee (IACUC).

## Author contributions

Conceptualization SV, NK-I, MES, ZGF and ZG; Methodology and investigation SV, NK-I, MES, AR, HA, AY, RA, AH, MBDN, MN, SH, VM, SJS, RD, ZH and JG; Supervision and writing original draft ZGF and ZG. All authors contributed to the article and approved the submitted version.

## Funding

ZG is supported by grants from the Israel Science Foundation (No. 756/15 and No. 405/18), Israel Cancer Association (20191819), the Israel Ministry of Science and Technology (Kamin, 59077), the Rosetrees Trust and the Israel Cancer Research Foundation (RCDA). ZGF - this research was partially supported by the Israel Science Foundation (ISF) - grant No. 1708/20, the Israel Ministry of Science and Technology (Kamin, 59078), the Sasson and Luiza Naor Fund, and the Israel Lung Association.

## Conflict of interest

ZGF and ZG are co-founders of Immunyx Pharma. MBDN, MN, SH and SJS are employees of Immunyx Pharma.

The remaining authors declare that the research was conducted in the absence of any commercial or financial relationships that could be construed as a potential conflict of interest.

## Publisher’s note

All claims expressed in this article are solely those of the authors and do not necessarily represent those of their affiliated organizations, or those of the publisher, the editors and the reviewers. Any product that may be evaluated in this article, or claim that may be made by its manufacturer, is not guaranteed or endorsed by the publisher.

## Supplementary material

Supplementary Figure 1 Validation of Phage Binding of Highest-Ranking Peptides Sequences Selected on Mouse Neutrophils. **(A)** Phage display panning for neutrophil specific peptides - The phage library was incubated with murine neutrophils to enrich neutrophil specific phages, binders were amplified and the amplificate was incubated with monocytes and lymphocytes for negative selection. After three rounds, enriched phages were subjected to sequencing. **(B, C)** Table **(B)** and pie diagram **(C)** of the top-ranking enriched peptide sequences and their prevalence in the final eluate (average of 5 replicates). **D.** WBC were isolated from 4T1 tumor bearing mice and binding of phage presenting the LQIQSWSSSP peptide to neutrophils (Ly6G^+^ CD11b^+^, red) and other WBC (Ly6G^-^CD11b^-^, blue) was evaluated using anti-M13 PE-labelled antibody and flow cytometry analysis. **(E)** Overview of LQI peptide constructs - the LQI peptide sequence on the phage is inserted within the protein VIII coat protein of the phage (LQI peptide in orange) and neighboring amino acid residues are depicted (recombinant LQI pVIII). An unlabeled, Cy5-labeled, biotin-labelled and the LQI peptide construct including neighboring amino acids from coat protein VIII as well as a glycine linker and fluorescein, were generated and tested for binding. **(F)** Competition assay of LQI phage binding and LQI peptide constructs. Neutrophils were incubated with the LQI-presenting phage (black line) alone or with the LQI-presenting phage and different LQI peptide constructs at the same time (blue line). Phage binding to neutrophils was quantified using anti-M13-PE antibody (only M13-PE = dotted line). **(H)** Representative image of confocal microscopy with Gr-1^+^ neutrophils (green) bound by the 16-LQI (red). SA=streptavidin.

Supplementary Figure 2 Effect of the LQI-tetramer on neutrophil function. **(A)** Neutrophils were incubated for 6.5 hours in the presence or absence of 10, 100 and 1000 nM of LQI tetramer. Treatment with 10 nM PMA was used as positive control. Viability was determined by trypan exclusion and compared to untreated control neutrophils. **(B, C)** FACS analysis of Annexin^+^ **(B)** and Annexin^+^PI^+^ **(C)** neutrophils following 30 min incubation in the presence or absence of 10, 100 and 1000 nM of LQI tetramer. Treatment with 10 nM PMA was used as positive control. **(D)** Mean fluorescence intensity (MFI) of CD11b expression in neutrophils following 30 min incubation in the presence or absence of 10, 100 and 1000 nM of LQI tetramer. Treatment with 10 nM PMA was used as positive control. **(E)** ROS production by neutrophils in the presence of absence of 10, 100 and 1000 nM of LQI tetramer. Treatment with 100 nM PMA was used as positive control. **(F, G)** Neutrophils migration (Boyden chamber) towards control medium or media containing LQI-tetramer 100 nM and 1 μM). Data represents average of 5 high power fields (HPF). **(H)** Neutrophils migration (Boyden chamber) towards control medium or media containing CXCL2 (100 ng/μl) alone or supplemented with LQI-tetramer (100 nM and 1 μM). The LQI-tetramer (100 nM and 1 μM LQI) was added to neutrophils in the upper chamber. Data represents average of 5 high power fields (HPF). The experiments were repeated at least 3 times with similar results. Error bars represent ± SEM.

Supplementary Figure 3 Optimization of LQI Tetramer Coating of PLGA NP. **(A)** FACS analysis of the binding of different PLGA nanoparticle formulations to neutrophils in whole blood. The top row plots neutrophils (SSC high) with nanoparticles fabricated from PLGA-PEG incubated with PBS (PBS-Cont., left), biotinylated LQI (LQI-Biotin, middle) or the LQI tetramer with c-terminal cysteine (LQI-SH). The top row plots neutrophils (SSC high) with nanoparticles fabricated from PLGA-PEG-Maleimide incubated with PBS (Mal-PBS-Cont., left), biotinylated LQI (Mal-LQI-Biotin, middle) or the LQI tetramer with c-terminal cysteine (Mal-LQI-SH). **(B)** MFI of PLGA-Cy5 for neutrophils and other WBC incubated with indicated NP formulations. **C.** Ratio of PLGA-Cy5 MFI between neutrophils and other WBC. **(D)** Binding of PLGA-PEG-Maleimide NP decorated with different concentrations of the LQI tetramer with c-terminal cysteine. **(E)** Electron microscopy images of uncoated NP and LQI tetramer coated NP.

Supplementary Figure 4 Effect of the KFP-tetramer on neutrophil function. **(A)** No effect of KFP-tetramer on neutrophil ROS production. **(B, C)** No effect of KFP-tetramer on neutrophil viability. **(D)** The KFP-tetramer is not chemoattractive to neutrophils. **(E)** No effect of KFP-tetramer on neutrophil attraction to CXCL2. The experiments were repeated at least 3 times with similar results. Error bars represent ± SEM.

Supplementary Table 1 Characteristics of human blood donors.

Movie S1 3-dimensional reconstitution of Z-stack confocal images of a neutrophil (green) and P-NP (white).
