# Supplementary figures and images for: Targeted nanoparticles modify neutrophil function in vivo
Source: Front Immunol. 2022 Oct 5;13:1003871. doi: 10.3389/fimmu.2022.1003871 (PMC9580275; doi:10.3389/fimmu.2022.1003871)

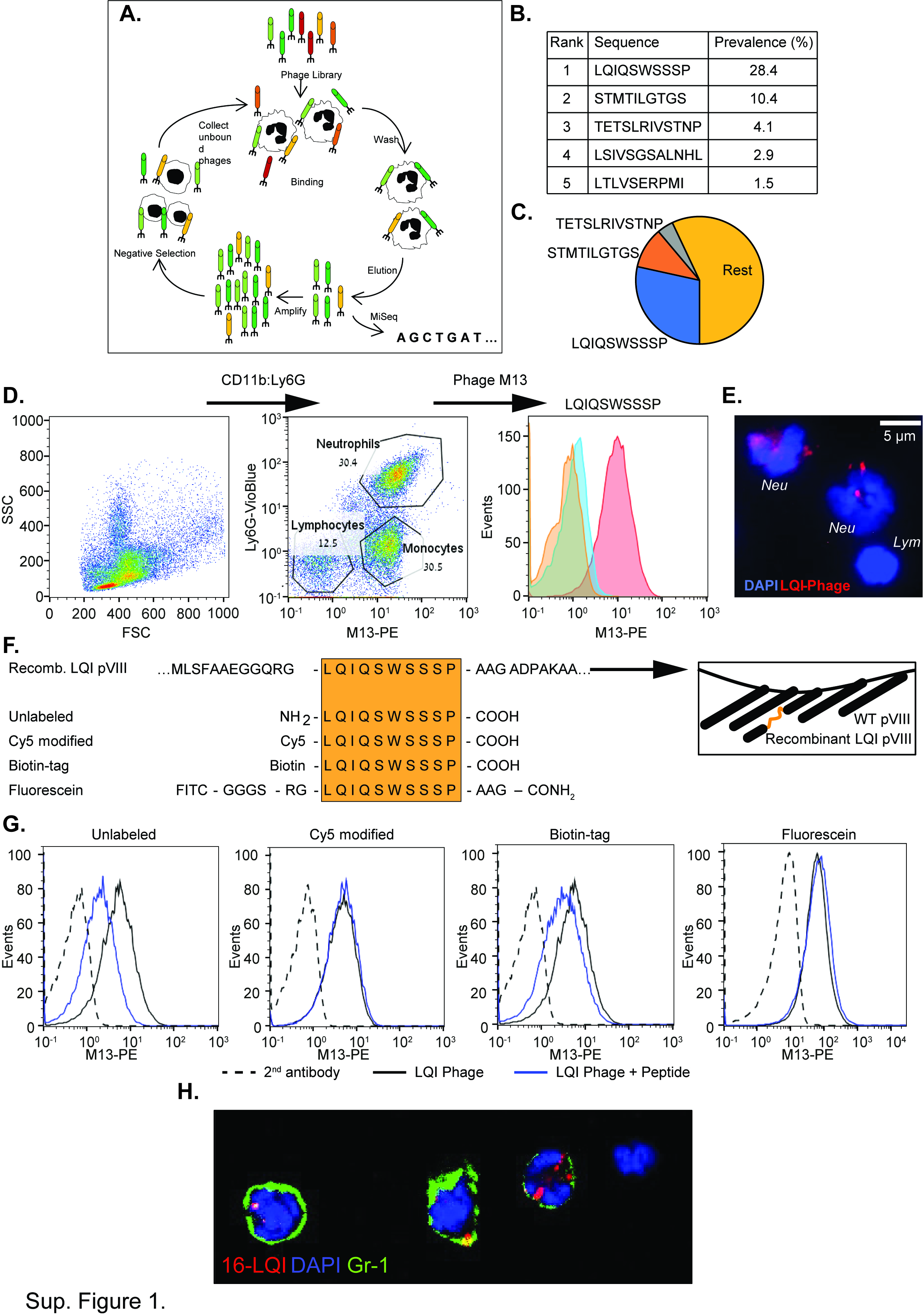

Supplement: Supplementary Figure 1 — Validation of Phage Binding of Highest-Ranking Peptides Sequences Selected on Mouse Neutrophils. (A) Phage display panning for neutrophil specific peptides - The phage library was incubated with murine neutrophils to enrich neutrophil specific phages, binders were amplified and the amplificate was incubated with monocytes and lymphocytes for negative selection. After three rounds, enriched phages were subjected to sequencing. (B, C) Table (B) and pie diagram (C) of the top-ranking enriched peptide sequences and their prevalence in the final eluate (average of 5 replicates). D. WBC were isolated from 4T1 tumor bearing mice and binding of phage presenting the LQIQSWSSSP peptide to neutrophils (Ly6G+ CD11b+, red) and other WBC (Ly6G-CD11b-, blue) was evaluated using anti-M13 PE-labelled antibody and flow cytometry analysis. (E) Overview of LQI peptide constructs - the LQI peptide sequence on the phage is inserted within the protein VIII coat protein of the phage (LQI peptide in orange) and neighboring amino acid residues are depicted (recombinant LQI pVIII). An unlabeled, Cy5-labeled, biotin-labelled and the LQI peptide construct including neighboring amino acids from coat protein VIII as well as a glycine linker and fluorescein, were generated and tested for binding. (F) Competition assay of LQI phage binding and LQI peptide constructs. Neutrophils were incubated with the LQI-presenting phage (black line) alone or with the LQI-presenting phage and different LQI peptide constructs at the same time (blue line). Phage binding to neutrophils was quantified using anti-M13-PE antibody (only M13-PE = dotted line). (H) Representative image of confocal microscopy with Gr-1+ neutrophils (green) bound by the 16-LQI (red). SA=streptavidin. [file Image_1.jpg]

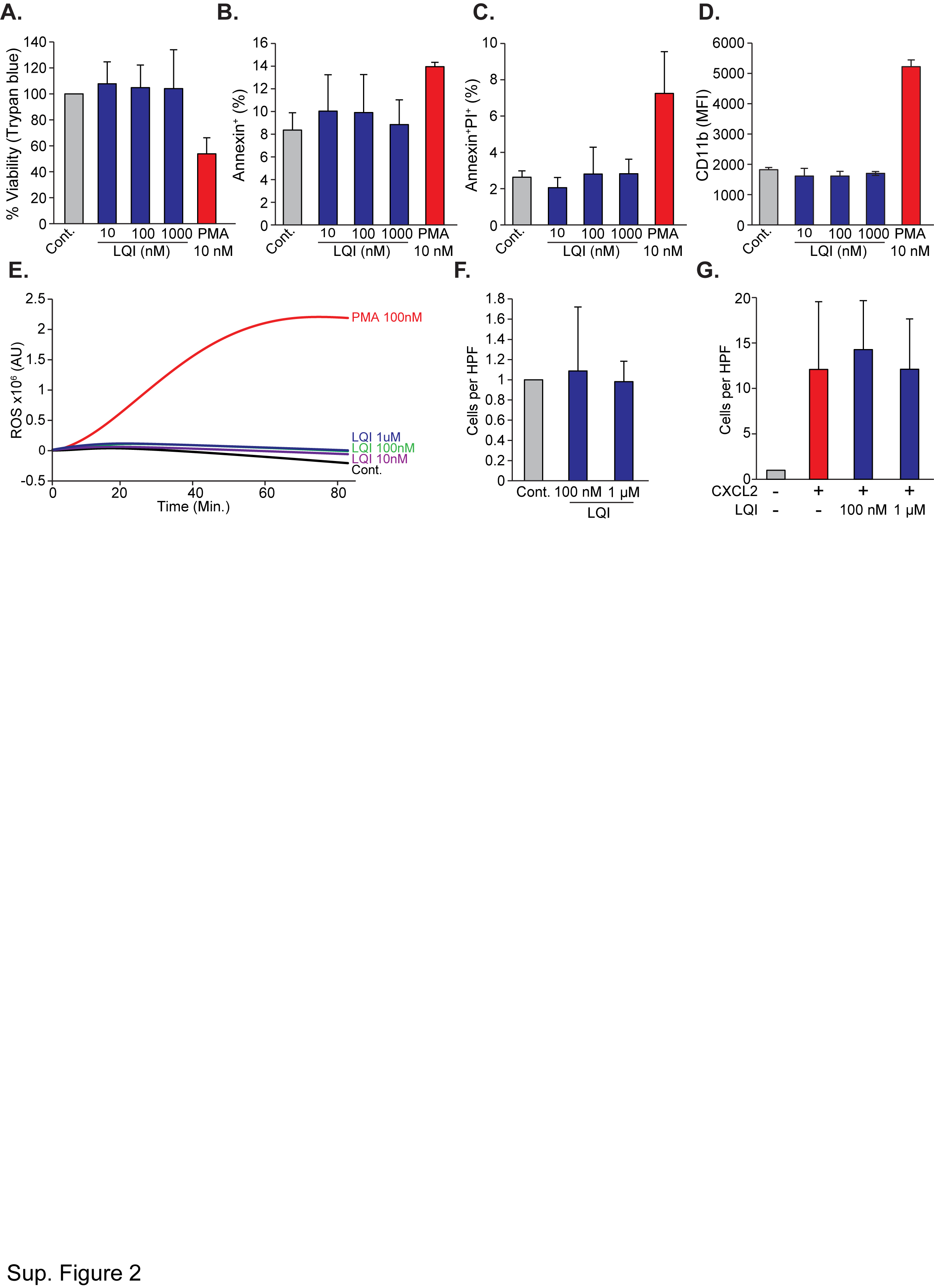

Supplement: Supplementary Figure 2 — Effect of the LQI-tetramer on neutrophil function. (A) Neutrophils were incubated for 6.5 hours in the presence or absence of 10, 100 and 1000 nM of LQI tetramer. Treatment with 10 nM PMA was used as positive control. Viability was determined by trypan exclusion and compared to untreated control neutrophils. (B, C) FACS analysis of Annexin+ (B) and Annexin+PI+ (C) neutrophils following 30 min incubation in the presence or absence of 10, 100 and 1000 nM of LQI tetramer. Treatment with 10 nM PMA was used as positive control. (D) Mean fluorescence intensity (MFI) of CD11b expression in neutrophils following 30 min incubation in the presence or absence of 10, 100 and 1000 nM of LQI tetramer. Treatment with 10 nM PMA was used as positive control. (E) ROS production by neutrophils in the presence of absence of 10, 100 and 1000 nM of LQI tetramer. Treatment with 100 nM PMA was used as positive control. (F, G) Neutrophils migration (Boyden chamber) towards control medium or media containing LQI-tetramer 100 nM and 1 μM). Data represents average of 5 high power fields (HPF). (H) Neutrophils migration (Boyden chamber) towards control medium or media containing CXCL2 (100 ng/μl) alone or supplemented with LQI-tetramer (100 nM and 1 μM). The LQI-tetramer (100 nM and 1 μM LQI) was added to neutrophils in the upper chamber. Data represents average of 5 high power fields (HPF). The experiments were repeated at least 3 times with similar results. Error bars represent ± SEM. [file Image_2.jpg]

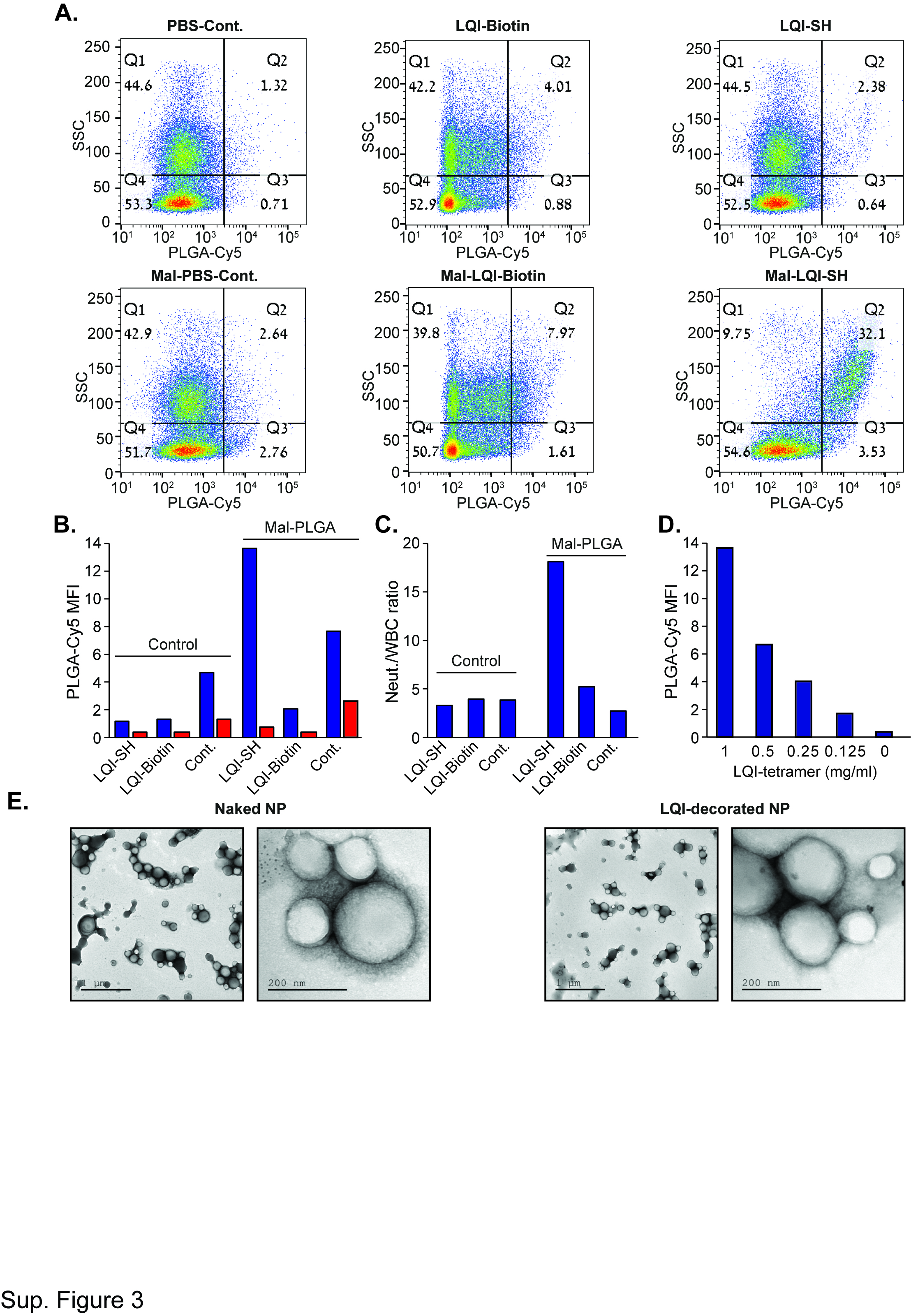

Supplement: Supplementary Figure 3 — Optimization of LQI Tetramer Coating of PLGA NP. (A) FACS analysis of the binding of different PLGA nanoparticle formulations to neutrophils in whole blood. The top row plots neutrophils (SSC high) with nanoparticles fabricated from PLGA-PEG incubated with PBS (PBS-Cont., left), biotinylated LQI (LQI-Biotin, middle) or the LQI tetramer with c-terminal cysteine (LQI-SH). The top row plots neutrophils (SSC high) with nanoparticles fabricated from PLGA-PEG-Maleimide incubated with PBS (Mal-PBS-Cont., left), biotinylated LQI (Mal-LQI-Biotin, middle) or the LQI tetramer with c-terminal cysteine (Mal-LQI-SH). (B) MFI of PLGA-Cy5 for neutrophils and other WBC incubated with indicated NP formulations. C. Ratio of PLGA-Cy5 MFI between neutrophils and other WBC. (D) Binding of PLGA-PEG-Maleimide NP decorated with different concentrations of the LQI tetramer with c-terminal cysteine. (E) Electron microscopy images of uncoated NP and LQI tetramer coated NP. [file Image_3.jpg]

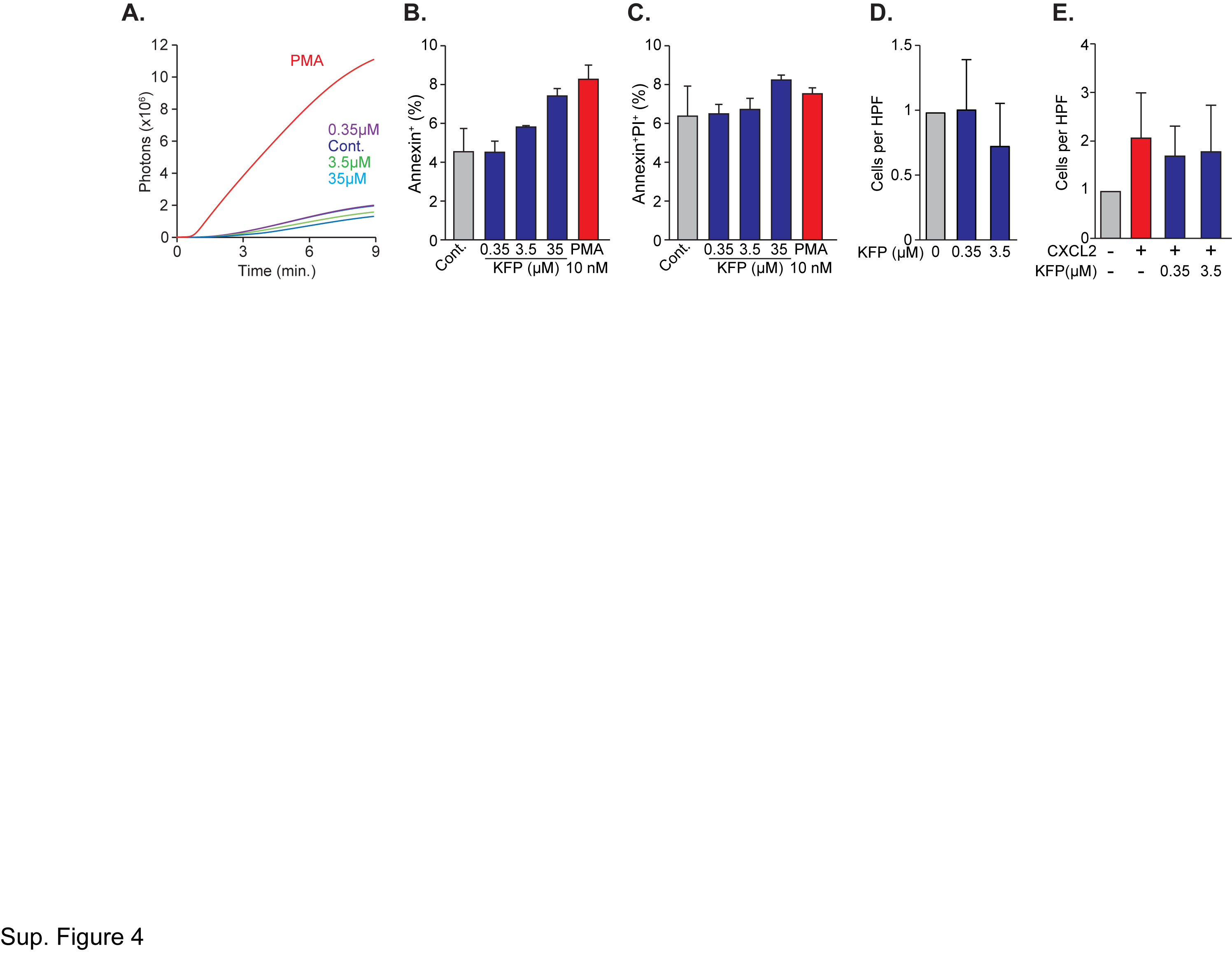

Supplement: Supplementary Figure 4 — Effect of the KFP-tetramer on neutrophil function. (A) No effect of KFP-tetramer on neutrophil ROS production. (B, C) No effect of KFP-tetramer on neutrophil viability. (D) The KFP-tetramer is not chemoattractive to neutrophils. (E) No effect of KFP-tetramer on neutrophil attraction to CXCL2. The experiments were repeated at least 3 times with similar results. Error bars represent ± SEM. [file Image_4.jpg]
