# Supplementary material for: Targeted nanoparticles modify neutrophil function in vivo
Source: Front Immunol. 2022 Oct 5;13:1003871. doi: 10.3389/fimmu.2022.1003871 (PMC9580275; doi:10.3389/fimmu.2022.1003871)
Supplement: Supplementary Table 1 — Characteristics of human blood donors. [file Table_1.pdf]

Donor	Age	F/M	Health status
**1**	33	F	Healthy
**2**	30	F	Healthy
**3**	60	F	Healthy
**4**	22	M	Healthy
**5**	45	M	Healthy
**6**	42	F	Healthy
**7**	48	M	Healthy
**8**	38	M	Healthy
**9**	25	F	Healthy
**10**	30	F	Healthy
**11**	57	F	Lung cancer
**12**	75	M	Stable COPD
**13**	81	M	Stable COPD

Sup. Table 1
